# Comparative assessment of breast volume using a smartphone device versus MRI

**DOI:** 10.1007/s12282-024-01647-6

**Published:** 2024-11-15

**Authors:** Annika S. Behrens, Hanna Huebner, Lothar Häberle, Marc Stamminger, Daniel Zint, Felix Heindl, Julius Emons, Carolin C. Hack, Naiba Nabieva, Michael Uder, Matthias Wetzl, Marius Wunderle, Matthias W. Beckmann, Peter A. Fasching, Sabine Ohlmeyer

**Affiliations:** 1 Department of Gynecology and Obstetrics, Comprehensive Cancer Center Erlangen-EMN (CCC ER-EMN), Erlangen University Hospital, Friedrich-Alexander-Universität Erlangen-Nürnberg, Erlangen, Germany; 2 Biostatistics Unit, Department of Gynecology and Obstetrics, Comprehensive Cancer Center Erlangen-EMN (CCC ER-EMN), Friedrich-Alexander-Universität Erlangen-Nürnberg, Erlangen, Germany; 3 Department of Computer Science, Visual Computing Lab, Friedrich-Alexander-Universität Erlangen-Nürnberg, Erlangen, Germany; 4 Department of Radiology, Erlangen University Hospital, Friedrich-Alexander-Universität Erlangen-Nürnberg, Erlangen, Germany; 5 Department of Gynecology, Erlangen University Hospital, University Breast Center for Franconia, Comprehensive Cancer Center Erlangen, European Metropolitan Area Nuremberg (CCC ER-EMN), Friedrich Alexander University of Erlangen-Nuremberg, Unversitätsstrasse 21-23, 91054 Erlangen, Germany

**Keywords:** Breast volume, MRI, Smartphone, Three-dimensional (3D)

## Abstract

**Background:**

Assessment of breast volume has a relevance for aesthetic surgery and for the prevention and prediction of breast diseases. This study investigated breast volume measurements using a three-dimensional (3D) body surface scanner integrated in a smartphone device in comparison with magnetic resonance imaging (MRI) scans.

**Methods:**

Breast volume was assessed for 22 women who underwent routine MRI imaging. 3D surface images were acquired using a smartphone’s digital texture camera (iPhone 11 Pro Max, Apple, California, USA, 2019).

Breast volumes were manually outlined and calculated by two independent investigators using a 3D software tool (Meshmixer 3.5, Autodesk, Inc., 2018). Volume assessments from MRI images were performed by a radiologist using Syngo.via (Siemens Healthcare, Erlangen, Germany, VB50). The agreement between both methods and the inter-observer agreement was calculated with the concordance correlation coefficients and analysed with Bland–Altman plots.

**Results:**

The mean breast volume as determined by MRI volumetry was 771.0 ml on the left side and 763.9 ml on the right side. Utilizing the 3D body surface volume assessment method, the mean breast volume was measured as 660.3 ml (observer A) and 616.8 ml (observer B) on the left side, and 701.9 ml (observer A) and 638.6 ml (observer B) on the right side. Although a high correlation was observed, differences in volume measurements appeared more pronounced in cases of larger breast volume.

**Conclusions:**

Smartphone-based 3D assessment of breast volume sufficiently agreed with MRI-based breast volume. This new technique could be used for cosmetic breast assessments in a surgical context and possibly in breast cancer risk studies assessing breast volume as outcome parameters.

## Introduction

Assessment of breast volume is of interest for several medical fields. It has been used to assess and predict cosmetic outcome following breast-conserving surgery precise planning for breast reduction surgery on the one hand and calculating breast implant sizes on the other hand [1–3].

A possible association between breast size and breast cancer risk has been discussed, studies reported controversial results [4–7]. Early detection of breast cancer and its preliminary stages is crucial with regard to therapeutic options and strategies [8, 9]. Overall, data on breast cancer risk estimation from breast volume are limited and warrants further assessment and clarification.

Potential associations between breast volume changes and the risk of breast cancer development later in life are yet to be determined. Changes in breast tissue and thereby in breast volume during lifetime occur due to various reasons such as pregnancy, breastfeeding, puberty and menopause, thus implicating hormonal influences [7, 10–17]. A recent observational study linked the signalling pathway of the serum receptor activator of nuclear factor kappa B ligand (RANKL) and its antagonist osteoprotegerin (OPG) to breast volume changes during pregnancy in healthy women, implicating potential alterations in breast cancer risk [18]. Routine use of breast volume measurements could provide a valuable addition to the understanding of hormonal changes throughout life. In combination with individual risk factors, it may even be possible to make predictions about a patient’s individual risk of developing breast cancer. However, in order to achieve this and to evaluate the possibility of a potential risk prediction, a better understanding and further large-scale studies are necessary.

However, breast volume assessment is not routinely performed in clinical practice, and methods are mostly experimental. To date, no validated standard method has been established.

Methods of breast volume assessment comprise various approaches, such as morphometric methods, calculating the breast volume from distances between defined points on the breast surface, or Archimedean methods based on Archimedes’ principle of water displacement, measuring the amount of water displaced by the breast [19–21]. Breast volume estimates have been calculated from mammograms [22, 23]. Other studies assessed the utility of a negative three-dimensional (3D) cast of the breast [24]. A systematic review from 2016 analysed various techniques focussing on accuracy [25]. Results showed the highest accuracy for magnetic resonance imaging (MRI)-based volume determination. Throughout the existing literature e.g. as described below, MRI imaging is often used as the gold standard reference method.

One promising approach to assess breast volume with reasonable accuracy using modern technology is calculating measurements on the basis of 3D imaging, such as stereo photography and laser scanning [26–28]. In a previous work, we assessed 3D surface-based volume measurements of the female breast with an optical 3D sensor as compared to MRI-based volume measures. We could show that this method was feasible, time-efficient and showed sufficiently accurate agreement with MRI-based breast volume [29].

However, most of these methods are costly, with the need of skilled personnel. Thus, the use of other optical methods of 3D surface imaging, e.g. widely available camera techniques appears to be a more cost-efficient and practicable approach. Recent studies assessed breast volume determination with 3D imaging using VECTRA XT 3D imaging system, for example with regard to plastic and aesthetic surgery with promising results [30–32].

MRI is a widely available, evidence-based and well-established imaging technique for breast tissue, however, breast volume measurements in MRI are performed for research purposes only [33, 34]. A systematic review deemed MRI volume measurement as the most accurate method of breast volumetry assessed [25]. Although MRI imaging of the breast is performed in clinical practice, it is only used in specific cases and only as complementary method to mammography and ultrasound imaging. Moreover, it is expensive. Thus, implementing MRI-based breast volume measurements for large cohorts does not seem to be feasible. In contrast, the use of a smartphone seems reasonable for several reasons. It is a mobile, easy-to-use method that is also widely available in the home environment. Breast examination by smartphone can be used as part of virtual or digital studies and thus pave the way for application of this method in the patients’ self-examination. In study settings, large cohorts can thus be reached without the participants having to appear at study centers. One recent study used a Laser Imaging Detection and Ranging (LiDAR) sensor on a smartphone to develop 3D breast scans and concluded that this technology could provide a low-cost and easy to use method with good accuracy [35].

Considering the potential fields of application of breast volumetry, it is important to establish time- and cost-efficient methods to assess breast volume. Moreover, with regard to breast volume changes, e.g. during pregnancy and potentially resulting implications for breast cancer risk calculations, establishing the use of non-radiation based, feasible and widely available assessment tools seems to be crucial.

In our current work, we studied the calculation of breast volume based on 3D body surface imaging performed with a smartphone device as compared to MRI imaging-based breast volumetry.

## Materials and methods

### Patients

Patients were recruited as a sub-project of the iMODE-B study (imaging and molecular detection of breast cancer). The cases included in this study were hospital-based. Imaging and data were retrieved consecutively from patients treated at the University Breast Center for Franconia, located in northern Bavaria (Germany), at the University of Erlangen-Nuremberg.

Between May 2020 and July 2020, 3D body surface assessment was obtained from 22 female patients. Patients had to be 18 years or older. We included patients with or without a history of breast cancer (see Table 1). Patients were eligible if they were scheduled for a standard-of-care MRI scan of the breast. 3D body surface assessment was performed at approximately the same time as MRI assessments (within a maximum of 6 weeks). Patients with a history of mastectomy or with a strong malformation of the breast (e.g. after extensive breast-conserving surgery or surgery with breast implants) were excluded.

**Table 1:** Characteristics of study population

Characteristic	Mean (SD)	*n* (%)
Age (years)	47.9 (12.3)	
BMI (kg/m^2^)	24.6 (5.6)	
Preexisting breast cancer		
No		12 (54.5)
Yes		10 (45.5)
Gravida		
0		10 (45.5)
Full-term pregnancies		
0		10 (45.4)
1		4 (18.2)
2		3 (13.6)
3		5 (22.7)

*SD* standard deviation, *BMI* body mass index

The study was approved by the Ethics Committee of the medical faculty at Friedrich Alexander University of Erlangen-Nuremberg, Erlangen, Germany (325_19 B). All investigations complied with national law and with the 1975 Declaration of Helsinki in its current revised version. All patients involved in the present work consented to imaging and inclusion in this trial and provided written consent.

### Data acquisition

All data concerning patient and tumor characteristics was documented conforming to the requirements of the German Cancer Society (*Deutsche Krebsgesellschaft*) and the German Society for Senology (*Deutsche Gesellschaft für Senologie*) as part of certification processes [36]. These requirements include histopathological tumor characteristics and epidemiological parameters. Clinical data were acquired as part of in-house routine medical history using standardized questionnaires.

### 3D body surface assessment

3D surface images were acquired via a digital texture camera of a smartphone (iPhone 11 Pro Max, Apple, California, USA, 2019) using a 3D imaging smartphone application (3d Scanner App™, Apple, California, USA, 2020).

**Fig. 1:**
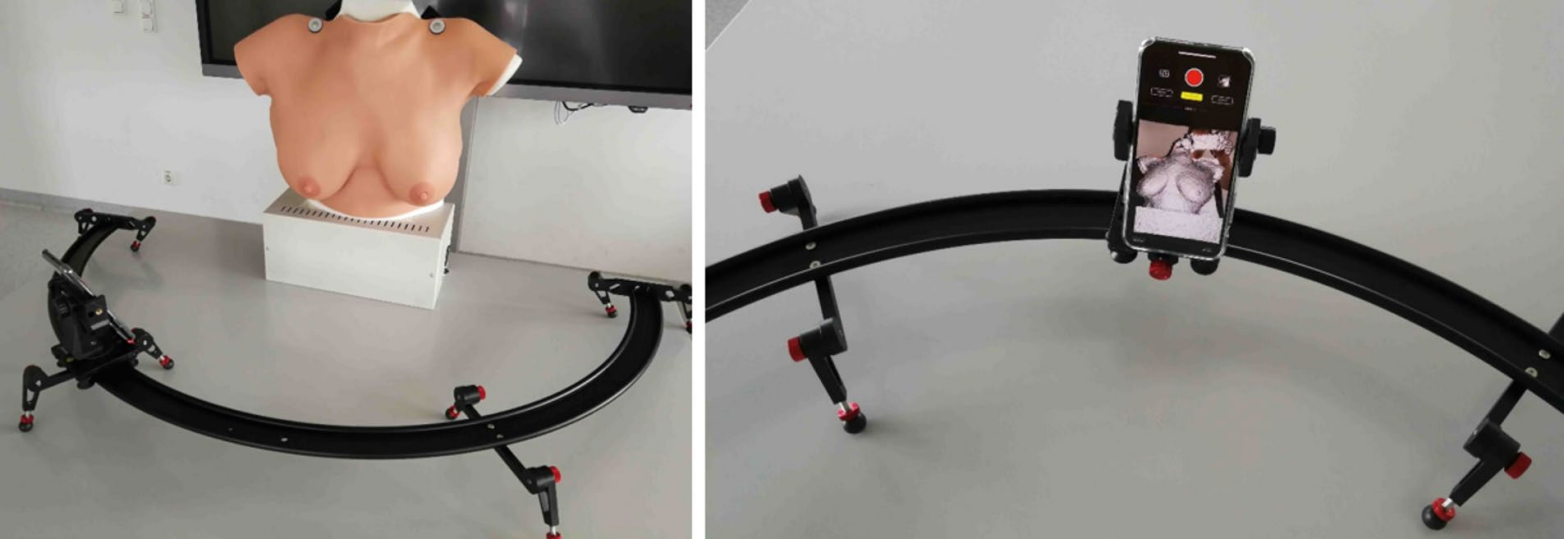
Setup of 3D images acquisition with an optical 3D sensor integrated in an iPhone 11 Pro Max. A metal rail was placed in front of the patient, containing a moveable slide for the iPhone. The metal rail was adjustable in height, the slide was moved manually and contained a 360° flexible gear for the fixation of the iPhone. Artificial breast model used for illustration

To eliminate potential influence of body position, etc., patients were instructed to stand in an upright position with the arms elevated by 90°, so both arms could form a horizontal line. 3D images were taken with an optical 3D sensor integrated in the abovementioned iPhone 11 Pro Max. A metal rail was placed in front of the patient, containing a moveable slide for the smartphone. The metal rail was adjustable in height, the slide was moved manually and contained a 360° flexible gear for the fixation of the smartphone. This system setup is depicted in Fig. 1.

For image acquisition, the True Depth sensor of the iPhone was placed on the gear upside down 5–10 cm below the submammary fold, in a distance of 30–35 cm to the thoracic wall and in a distance of at least 20 cm to the mamilla. Assessment started in a 45° angle relative to the patient capturing mainly the right breast and partly the left. The smartphone was moved on the rail until the posterior axillary line of the left breast was captured and was then moved back to the posterior axillary line of the right breast.

### 3D image volumetry

The acquired scans were output from the iPhone scanner in a Polygon File Format (PLY). The following data processing steps were each carried out by two independent investigators (M.W., “observer A” and A.S.B. “observer B”). The scanning of the dataset for volumetry is performed in about 1 min and a half acquisition time. The PLY files generated from the iPhone scanner were imported into MeshLab (MeshLab 2020.12, ISTI-CNR, Italy, 2020). This software was used to reduce data size and repair rough defects in the generated 3D models. Afterwards, the corrected files were converted into OBJ files and were further processed using a 3D software tool (Meshmixer 3.5, Autodesk, Inc., 2018). Further closing of smaller defects in the surface of the 3D models was carried out. This step was crucial for volume calculation within the program. The region of interest for volume assessment was indicated by colored markings and comprised the total breast tissue of each breast, defined by the superior, superolateral, medial, inferior and lateral breast boundaries. The breast was virtually removed from the 3D model using the sculpting tools of Meshmixer. The volume of the union of the 3D model before and after sculpting represented the breast volume and could be calculated directly in Meshmixer. Breast volume was calculated from both breasts for each patient.

### MRI volumetry

MRI scans were performed using a 3.0 Tesla MRI unit (MAGNETOM Vida; Siemens Healthcare, Erlangen, Germany) with a dedicated 16-channel bilateral breast coil (16-Channel AI Breast Coil; Siemens Healthcare, Erlangen, Germany). Patients were placed in prone position with the breasts immobilized using foamed material, without compression. For breast volumetry, a non-enhanced T1w 3D dataset was used (Image parameters: T1w Dixon in transversal orientation, TR 5.41 ms, TE 2.46 ms, Voxel size 0.8 × 0.8 × 1.5 mm^3^, FoV 380 × 380, Matrix 448 × 358, Slice thickness 1.5 mm, 112 Slices, GRAPPA × 3, Bandwidth 859 Hz/pixel, Acquisition time 1:08 min).

The calculation of the breast volumes was performed on T1w dixon in-phase images by a board-certified radiologist using a semiautomatic, threshold-based workflow of an analysis software (MM Reading, Syngo.Via; Siemens Healthcare, Erlangen, Germany). In the first step, the dataset was loaded to the image segments of the 3D workflow platform in multimodality reading (MM) Reading. Segmentation was initialized by the region growing tool. The Volume Rendering Technique punch tool with the option “Remove Outside” was selected and a coarse segmentation of the breast on the 3D rendering view was drawn manually. This was repeated on coronal and sagittal orientations. The lower threshold was set from 0 to a maximum of 50, with the aim that the volume drawn in color by the algorithm most closely corresponds to the actual volume of the breast. After that, final details were corrected using the FreeHand MPR (Multiplanar Reformation) Tool, if necessary. This occurred most commonly in the plane of the pectoral fascia. The breast itself was defined to include the areola, nipple, and outer surface skin as the surface limit and the pectoral muscle (excluded from volume) as the dorsal limit. The medial parasternal limit was identified, and the lateral border was defined as this point of the lateral thorax wall, where the subcutaneous fat of the breast reached the same height as the subcutaneous fat of the thorax wall. The final colored volume was checked on every transversal image using the 3D mode again. The overall breast volume of each breast was then automatically calculated by the analysis software in ml. The volume segmentation for each breast required approximately 5 min.

### Statistical analysis

This study aimed to assess the agreement between the 3D body surface method and MRI volumetry to determine breast volume. Furthermore, the inter-observer agreement was assessed using the 3D body surface method performed by two independent observers.

The agreement between measurement methods and the inter-observer agreement was assessed with the concordance correlation coefficients (CCC) and analysed with Bland–Altman plots [37, 38]. The CCC ranges from −1 (perfect reversed agreement) to 0 (no agreement at all) to 1 (perfect agreement). With Bland–Altman plots, the average of both observers’ measurements is plotted against the difference in the measurements. The mean difference and the “limits of agreement” (mean difference plus/minus 1.96 times standard deviation) are calculated and displayed. If the differences are normally distributed, 95% of the differences will lie between these limits.

Those measures were calculated for both observers and both breast sides. The 95% confidence interval (CI) to each measure was also calculated.

The calculations were carried out using the R system for statistical computing (version 3.6.1; R Development Core Team, Vienna, Austria, 2019).

## Results

The mean age of the patients was 48 years (range 27–79 years), and the mean body mass index was 24.6 kg/m^2^ (range 18.5–39.1 kg/m^2^). The patient characteristics are presented in Table 1. 22 patients were included in the final analysis.

**Fig. 2:**
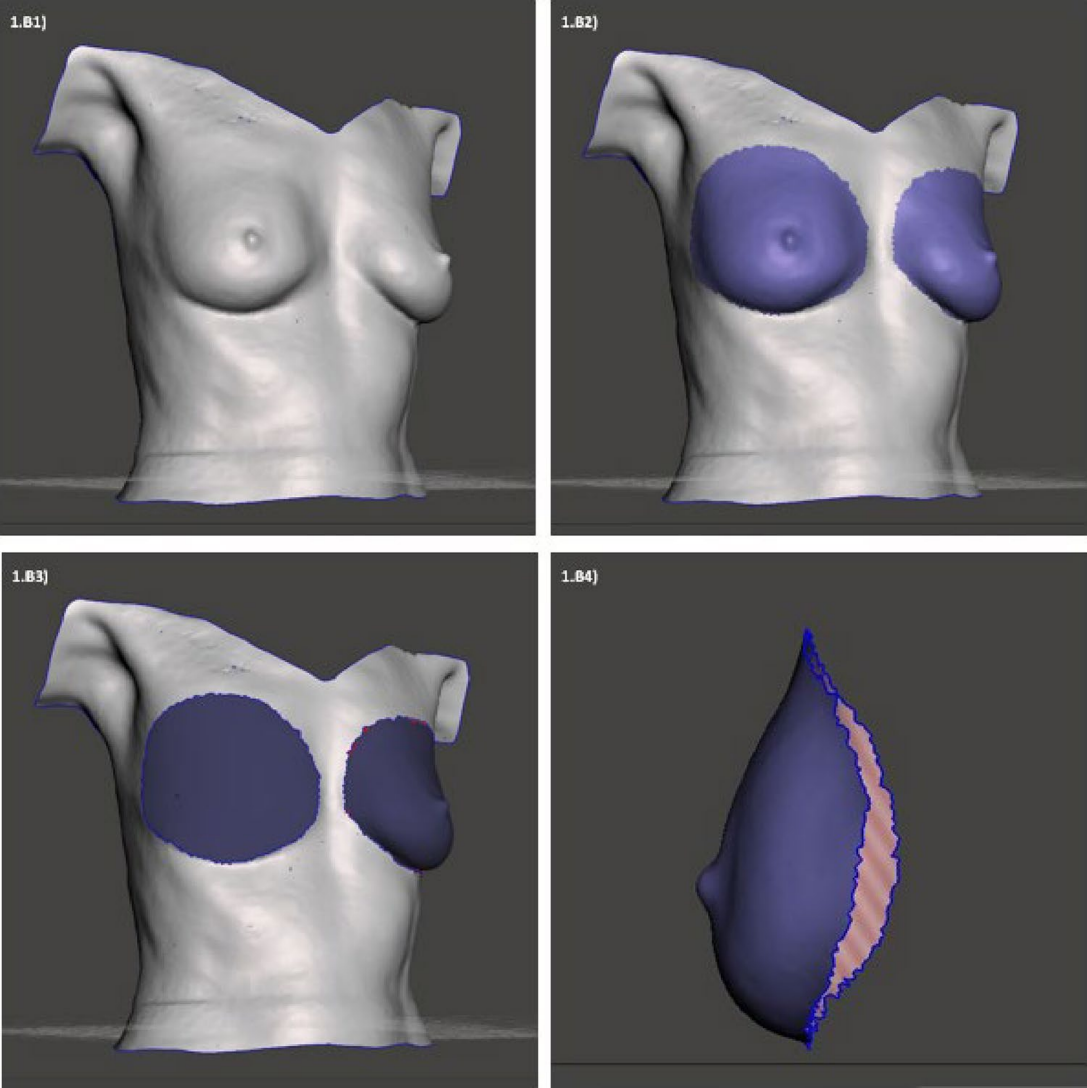
Example of three-dimensional (3D) body surface volume measurement using a 3D software tool (Meshmixer 3.5). (1) Original 3D image after performing corrective adjustments as indicated after image conversion from Meshlab. (2) Colored marking of breast area eligible for volume calculation. (3) Depiction of thoracic wall area of the right breast. (4) Breast computation for volume calculation

**Fig. 3:**
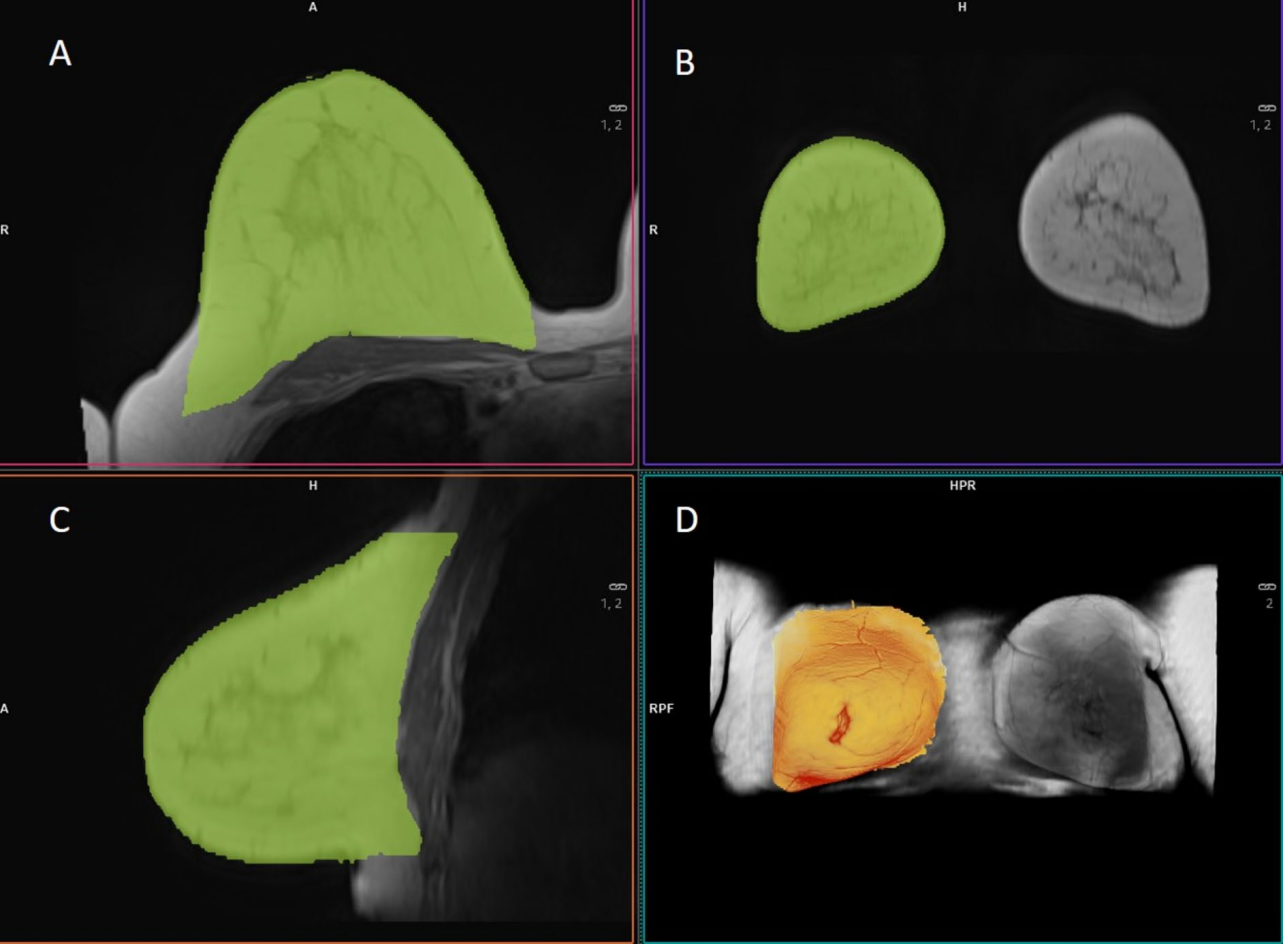
Example of semiautomatic MRI volumetry (MM Reading, Syngo.via, Siemens Healthcare, Erlangen, Germany). **A** Transversal, **B** Coronal, **C** sagittal slice orientation of semiautomatic volumetry of the right breast. **D** Corresponding VRT dataset in head-feet orientation

An example of 3D body surface volume measurements as performed with Meshmixer 3.5 is depicted in Fig. 2, an example for MRI volume measurement is shown in Fig. 3.

**Fig. 4:**
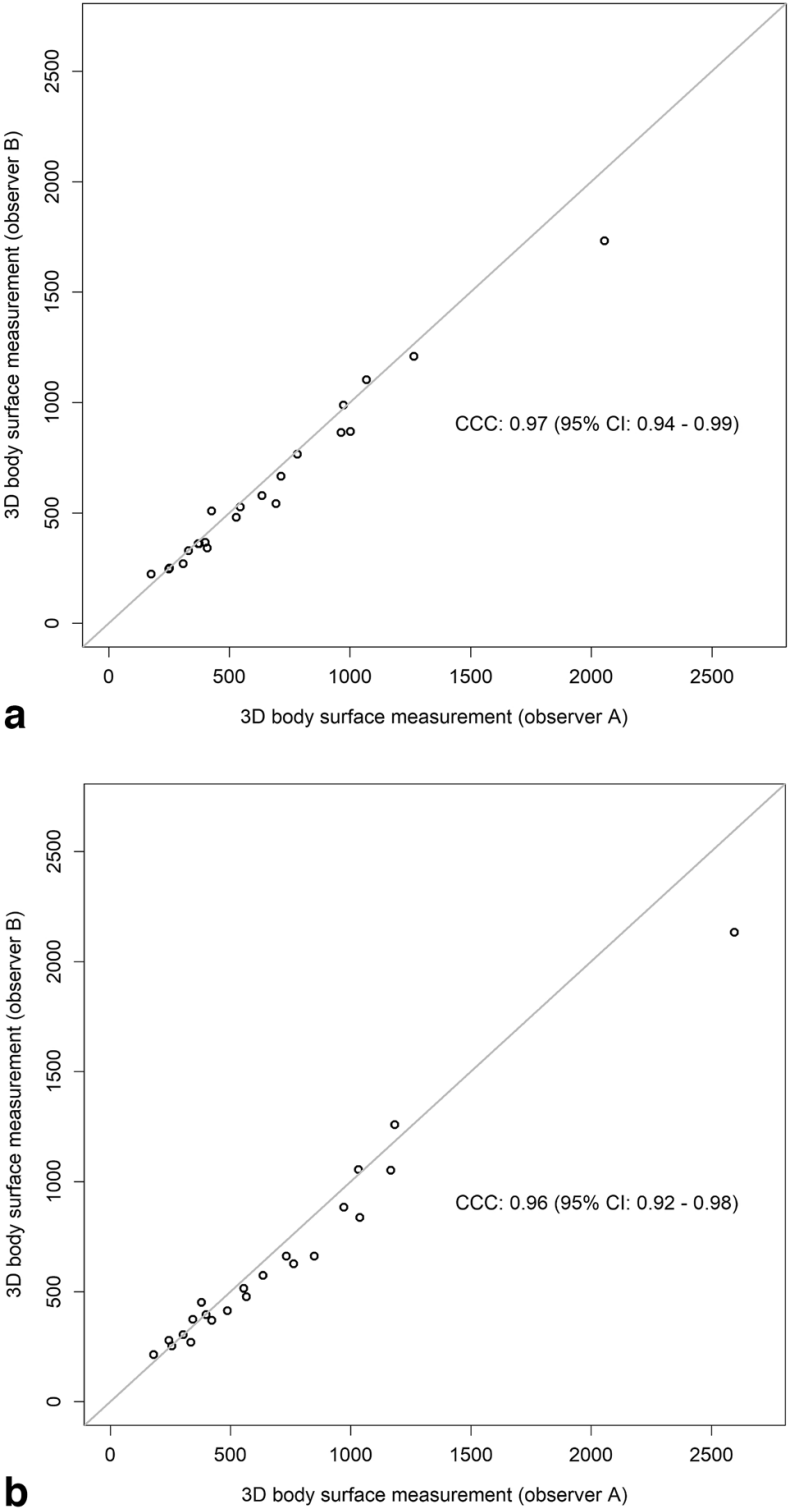
Scatter plot for 3D body surface volume measurements of the left (**A**) and right (**B**) breasts by two observers

Table 2 shows results of the 3D body surface volume measurements, the inter-observer agreement separately for the left and right breast as well as the agreement between 3D body surface volume measurement separated for observer A and B and MRI volume measurement. The mean difference between measurements performed by observer A and B were 43.5 ml (95% CI: 6.79; 80.21) for the left breast and 63.23 ml (95% CI: 11.41; 115.05) for the right breast. The CCC showed substantial agreement (left breast: 0.97 (0.94, 0.99); right breast: 0.96 (0.92, 0.98)). While agreement between MRI and observer A 3D image volumetry was substantial (left breast: 0.9 (0.89, 0.97); right breast: 0.98 (0.95, 0.99)), agreement between MRI and observer B were modest to poor (left breast: 0.87 (0.75, 0.93); right breast: 0.93 (0.86, 0.97)). Corresponding scatter plots for 3D body surface volume measurements of the left (A) and right (B) breasts by two observers are shown in Fig. 4 while according Bland–Altman plots are shown in supplementary material.

**Table 2:** Agreement between three-dimensional (3D) body surface volume measurement MRI volume measurement

	Left breast in ml (95% CI)	Right breast in ml (95% CI)
*Mean 3D volume measurement*		
Observer A	660.32 (467.13, 853.50)	701.86 (468.49, 935.23)
Observer B	616.82 (447.42, 786.21)	638.64 (442.63, 834.64)
*Mean MRI volume measurement*	771.00 (543.73, 998.27)	763.91 (534.02, 993.80)
*Mean difference*		
Between observer A and B	43.50 (6.79, 80.21)	63.23 (11.41, 115.05)
Upper limit of agreement	205.79 (142.20, 269.37)	292.30 (202.55, 382.06)
Lower limit of agreement	−118.79 (−182.37, −55.20)	−165.85 (−255.60, −76.09)
CCC	0.97 (0.94, 0.99)	0.96 (0.92, 0.98)
Between observer A and MRI	−110.68 (−161.64, −59.72)	−62.05 (−100.20, −23.89)
Upper limit of agreement	114.59 (26.33, 202.86)	106.62 (40.54, 172.71)
Lower limit of agreement	−335.96 (−424.22, −247.69)	−230.71 (−296.80, −164.63)
CCC	0.95 (0.89, 0.97)	0.98 (0.95, 0.99)
Between observer B and MRI	−154.18 (−234.84, −73.52)	−125.27 (−181.53, −69.02)
Upper limit of agreement	202.37 (62.67, 342.08)	123.40 (25.97, 220.84)
Lower limit of agreement	−510.74 (−650.44, −371.03)	−373.95 (−471.38, −276.51)
CCC	0.87 (0.75, 0.93)	0.93 (0.86, 0.97)

*CI* confidence interval, *CCC* concordance correlation coefficient

**Fig. 5:**
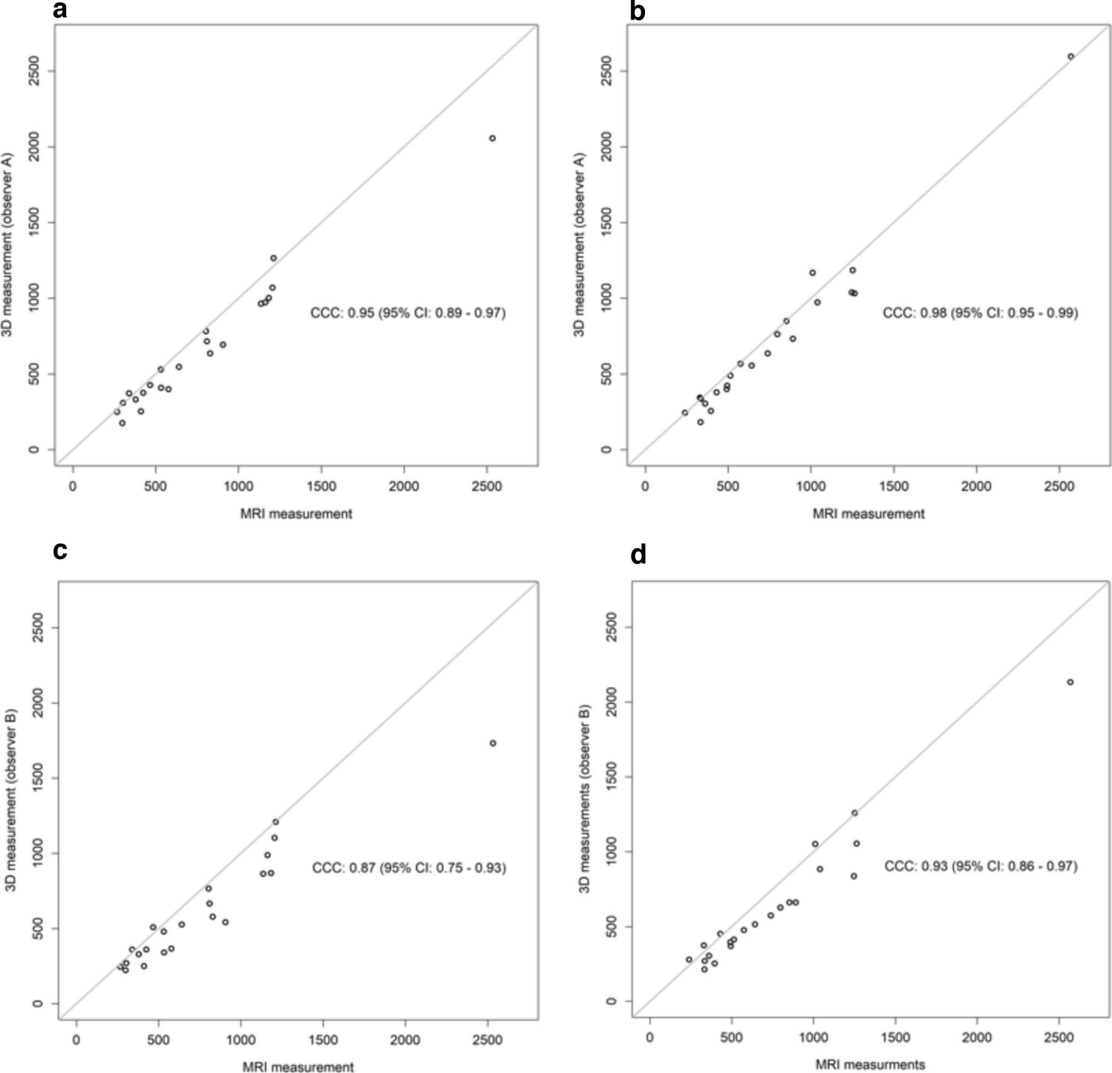
Scatter plots for comparison of methods. MRI and 3D body surface volume measurements by observer A of the **a** left breast and **b** right breast and by observer B of the **c** left breast and **d** right breast

**Fig. 6:**
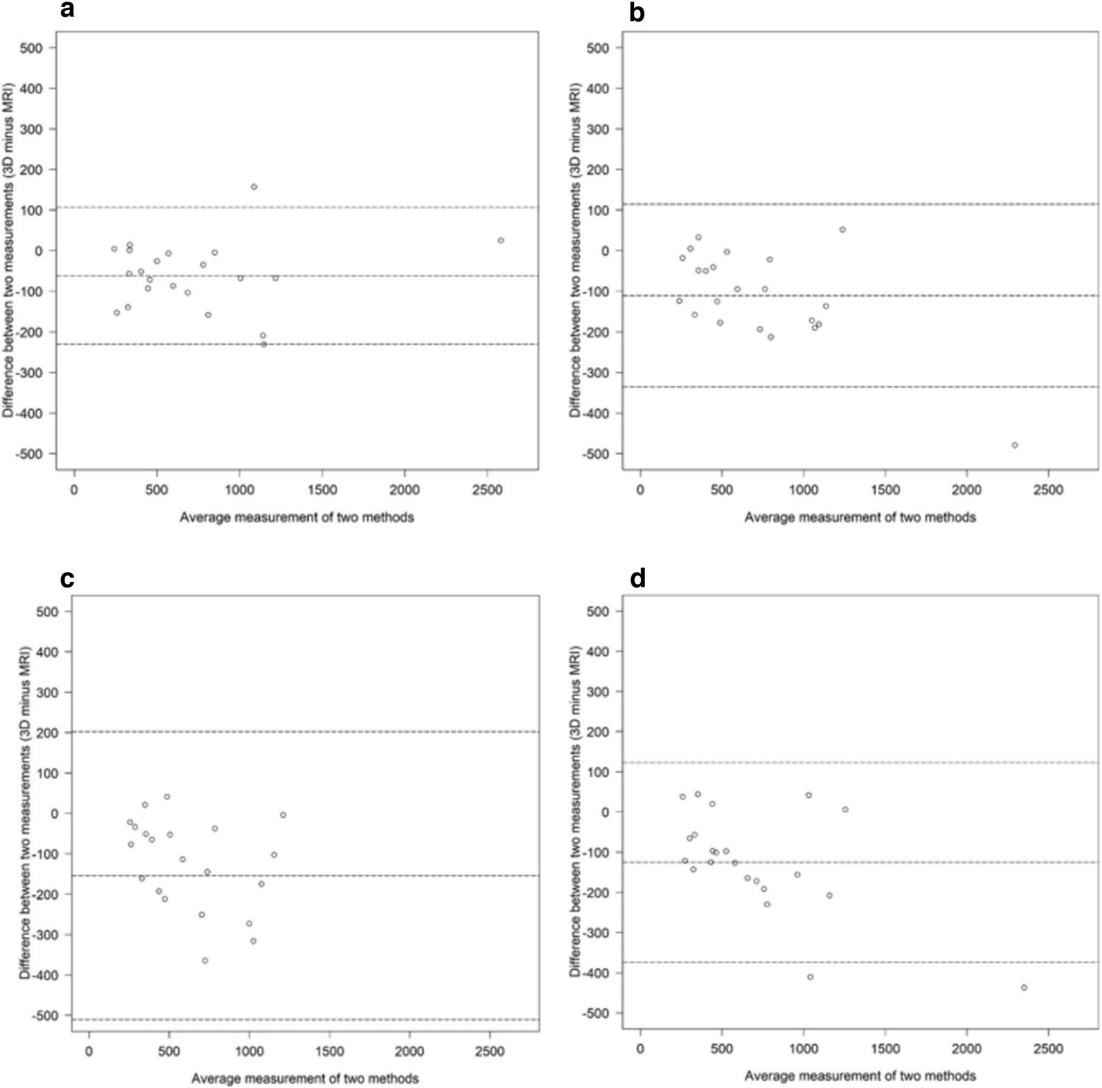
Bland–Altman plots for comparison of methods. MRI and 3D body surface volume measurements by observer A of the **a** left breast and **b** right breast and by observer B of the **c** left breast and **d** right breast. The middle dashed line indicates the mean difference. The upper and lower dashed lines display “limits of agreement”

Scatter plots for comparison of methods between MRI and 3D body surface volume measurements of the left breast by (a) observer A and (b) observer B and those measurements of the right breast by (c) observer A and (d) observer B are shown in Fig. 5 while Bland–Altman plots for comparison of methods are shown in Fig. 6.

## Discussion

The present work showed, that capturing 3D volumes with a smartphone device is feasible and results in reasonably accurate volume assessments. Inter-observer agreement of 3D body surface volume measurement was satisfactory. Agreement of 3D body surface volume measurement and MRI volume measurement was modest, indicating method-depending results. As described in our previous study, one potential source of inaccuracy is the definition of the dorsal border of the breast. 3D body surface volume measurement do not allow visibility of the dorsal limit, as opposed to MRI imaging [29]. This potential inaccuracy can also be transferred to the differences in concordance rates between observers, as the manual border was outlined manually. However, differences between methods appeared to be smaller than in our previous study. This could be because of different imaging and calculation methods and softwares. Overall, volumes as assessed via MRI were greater than the corresponding 3D body surface measurements. Patients with a higher breast volume appeared to be more prone to discrepancy in measurements. Our results are in concordance with similar studies [29–31]. For future trials, a larger number of patients should be evaluated.

Imaging of the breast using a smartphone device is advantageous with regard to availability and time-efficiency for both the observer and the patient. It is comparatively easy to operate, no specifically trained personnel is needed. Material costs are low compared to other commercially available 3D assessment devices. However, data processing of the acquired records is rather elaborate. The use of different tools such as MeshLab and Meshmixer is required for which training is essential. Other smartphone devices and applications such laser imaging detection and ranging (LiDAR) are subject to several clinical trials. One study used this method to assess geometric measurements of the breast [35]. However, they did not determine breast volumes. Moreover, LiDAR sensors are, to date, not standard equipment of smart phone devices.

Defects within the mesh, particularly in the inframammary fold could potentially lead to over- or underestimation of the breast volume due to the required manual correction. Future studies could aim to minimize this limitation, e.g. by using other devices. Moreover, parameters such as patient posing could potentially inflict volume measurement results [39]. In the current work, we aimed to minimize this potential bias by standardizing assessments in an upright position with arms elevated at a 90° angle.

In contrast to the 3D body surface method, MRI scans are too expensive to be performed routinely for volume measurement and scan time is limited [40]. In our opinion, the written informed consent, the changeover time between two women and the correct positioning take the most time here, three factors that cannot be accelerated significantly. The scanning of the dataset for volumetry itself is not the limiting component with about 1 min and a half acquisition time. Nevertheless, there are certain differences between the 3D surface method and MRI volumetry. Because the scan is performed with the woman in a prone position, the anatomy of the breast deviates from the anatomy in an upright position. In contrast to the 3D surface method, the anatomy can be displayed directly. Nevertheless, there is a certain variance when determining the dorsal boundary of the breast and the exact boundaries must be defined for use in clinical routine [25].

Breast volume assessments are of interest for several medical scopes such as assessment and prediction of cosmetic outcomes following breast-conserving oncologic surgery or aesthetic surgery, e.g. in planning pre- and post-surgical volume estimates and other aspects of surgery to achieve cosmetically satisfying results according to the patient wishes. Measurements could for example be used for pre- and postoperative evaluation after oncologic surgery such as partial mastectomies.

With regard to breast volume changes, e.g. during pregnancy and potentially resulting implications for breast cancer risk calculations, establishing the use of non-radiation based, feasible and widely available assessment tools seems to be crucial. 3D imaging of the breast is a widely researched topic with great potential. With regard to future developments in medical healthcare, establishing methods with the potential to allow home-based self-assessment is of interest. The use of smartphone devices could potentially be extended to such self-assessments, with possible regard to individualized risk assessment and breast cancer prevention. It is a mobile, easy-to-use method that is also widely available in the home environment. Thus, access to medical care and cancer prevention could be largely improved, especially in structurally weak areas, rural areas or for patients with limited mobility. In addition, this could relieve and improve the appointment process at specialized centers, which is often affected by long waiting times.

## Conclusions

Smart phone-based 3D assessment of breast volume sufficiently agreed with MRI-based breast volume. This new technique could be used in large-scale epidemiologic studies such as breast cancer risk studies assessing breast volume as an outcome parameter. Breast examination by smartphone could be used as part of virtual or digital studies and thus pave the way for application of this method in the patients’ self-examination. Another potential application field could lie in cosmetic breast assessments in a surgical context. The use of smartphone devices is a low-cost, mobile, easy-to-use method that is also widely available in the home environment.

## Data Availability

Additional datasets used and/or analysed during the current study are available from the corresponding author upon reasonable request.
