# Correction: Comparative assessment of breast volume using a smartphone device versus MRI

**DOI:** 10.1007/s12282-025-01685-8

**Published:** 2025-03-01

**Authors:** Annika S. Behrens, Hanna Huebner, Lothar Häberle, Marc Stamminger, Daniel Zint, Felix Heindl, Julius Emons, Carolin C. Hack, Naiba Nabieva, Michael Uder, Matthias Wetzl, Marius Wunderle, Matthias W. Beckmann, Peter A. Fasching, Sabine Ohlmeyer

**Affiliations:** 1 Department of Gynecology and Obstetrics, Comprehensive Cancer Center Erlangen-EMN (CCC ER-EMN), Erlangen University Hospital, Friedrich-Alexander-Universität Erlangen-Nürnberg, Erlangen, Germany; 2 Biostatistics Unit, Department of Gynecology and Obstetrics, Comprehensive Cancer Center Erlangen-EMN (CCC ER-EMN), Friedrich-Alexander-Universität Erlangen-Nürnberg, Erlangen, Germany; 3 Department of Computer Science, Visual Computing Lab, Friedrich-Alexander-Universität Erlangen-Nürnberg, Erlangen, Germany; 4 Department of Radiology, Erlangen University Hospital, Friedrich-Alexander-Universität Erlangen-Nürnberg, Erlangen, Germany; 5 Department of Gynecology, Erlangen University Hospital, University Breast Center for Franconia, Comprehensive Cancer Center Erlangen, European Metropolitan Area Nuremberg (CCC ER-EMN), Friedrich Alexander University of Erlangen-Nuremberg, Unversitätsstrasse 21-23, 91054 Erlangen, Germany

**Correction: Breast Cancer (2024) 32:166-176** 10.1007/s12282-024-01647-6

The article ‘Comparative assessment of breast volume using a smartphone device versus MRI’, written by Annika S. Behrens, Hanna Huebner, Lothar Häberle, Marc Stamminger, Daniel Zint, Felix Heindl, Julius Emons, Carolin C. Hack, Naiba Nabieva, Michael Uder, Matthias Wetzl, Marius Wunderle, Matthias W. Beckmann, Peter A. Fasching, Sabine Ohlmeyer, was originally published under exclusive license to The Author(s), under exclusive licence to The Japanese Breast Cancer Society. As a result of the subsequent decision to publish the article under the open access model, the article’s copyright notice was changed on 12 February 2025 to © The Author(s) 2025 and the article is now distributed under a Creative Commons Attribution 4.0 International License, which permits use, sharing, adaptation, distribution and reproduction in any medium or format, as long as you give appropriate credit to the original author(s) and the source, provide a link to the Creative Commons licence, and indicate if changes were made. The images or other third party material in this article are included in the article’s Creative Commons licence, unless indicated otherwise in a credit line to the material. If material is not included in the article’s Creative Commons licence and your intended use is not permitted by statutory regulation or exceeds the permitted use, you will need to obtain permission directly from the copyright holder. To view a copy of this licence, visit http://creativecommons.org/licenses/by/4.0/.

The original article has been corrected.

